# Supplementary material for: Spin-glass-like freezing of inner and outer surface layers in hollow γ-Fe2O3 nanoparticles
Source: Sci Rep. 2015 Oct 27;5:15054. doi: 10.1038/srep15054 (PMC4621521; doi:10.1038/srep15054)
Supplement: Supplementary Information [file srep15054-s1.pdf]

Spin-glass-like freezing of inner and outer surface layers in hollow γ-Fe_2O_3 nanoparticles

Supplementary Information

Hafsa Khurshid[1,*], Paula Lampen-Kelley[1,*], Òscar Iglesias[2], Javier Alonso[1,3], Manh-Huong Phan[1,*], Cheng-Jun Sun[4], Marie-Louise Saboungi[3,5] , and Hariharan Srikanth[1,*]

[1] *Department of Physics, University of South Florida, Tampa, FL 33620*
[2] *Departament de Física Fonamental and Institut de Nanociència i Nanotecnologia (In[2]UB) Universitat de Barcelona, Av. Diagonal 647, 08028 Barcelona, Spain*
[3] *BCMaterials Edificio No. 500, Parque Tecnológico de Vizcaya, Derio, Spain 48160*

[4] *Advanced Photon Source, Argonne National Laboratory, Argonne, Illinois 60439*
[5] *IMPMC-Université Pierre et Marie Curie F-75252 Paris- Cedex 5 & Université d'Orléans*

* Corresponding authors: khurshid@usf.edu; plampen@utk.edu; phanm@usf.edu;

sharihar@usf.edu

S.1 Dipolar interactions

The strength of dipolar interactions in our system can be estimated using the following expression:

$$E_{dip} = \frac{\mu_0 \mu^2}{4\pi L^3}. \qquad (1)$$

The magnetic moment μ can be estimated from the fitting of the room temperature hysteresis loop to a Langevin function (see Fig. S.1), yielding a magnetic moment of $\mu \sim 1.03\times10^{-20}$ Am^2/particle. Since the nanoparticles in our study are packed together, L can be approximated as the diameter of the particle (14.7 nm). This yields a corresponding dipolar temperature $T_{dip} = E_{dip}/k_B \sim 0.24$ K, indicating that dipolar interparticle interactions are negligible.

Alternatively, since we note that the above argument does not take into account the hollow polycrystalline geometry of the particles, we can estimate the maximum interaction between two grains in adjacent particles. In this case we take the separation to be equal to the shell thickness (L = 3.25 nm) and μ to be 25% of the total particle moment based on the number of grains (~4-6) visible in the HRTEM images of Fig. 1. The resulting dipolar temperature of T_{dip} = 1.4 K in this approach is still insignificant compared to the experimentally observed onset of slow dynamics.

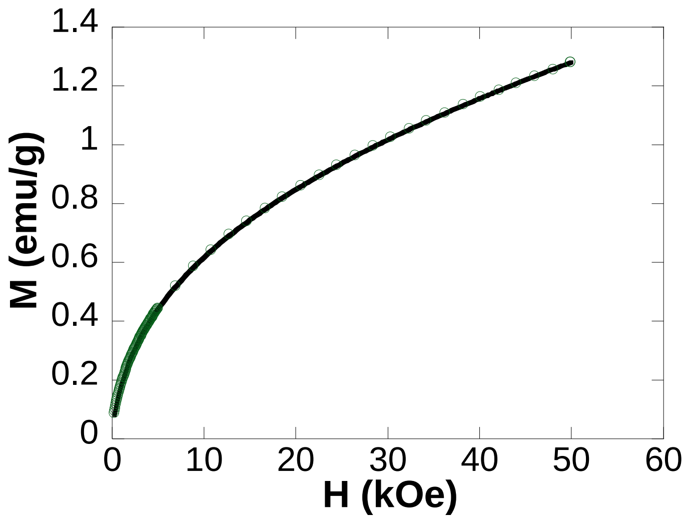

Fig. S.1 Room temperature magnetization curve of 15 nm γ-Fe_2O_3 hollow particles. Solid line represents best fit to a Langevin function.

Finally, dispersion of the particles in a wax medium to systematically control particle concentration showed no significant effect on the peak location in the zero-field-cooled M (T) curves within experimental error. The curves for the packed powder sample and a 15 wt.%

dispersion are shown in Fig. S.2. This observation confirms that interparticle interactions in our system can be neglected and the behavior of the system is dominated by intraparticle effects.

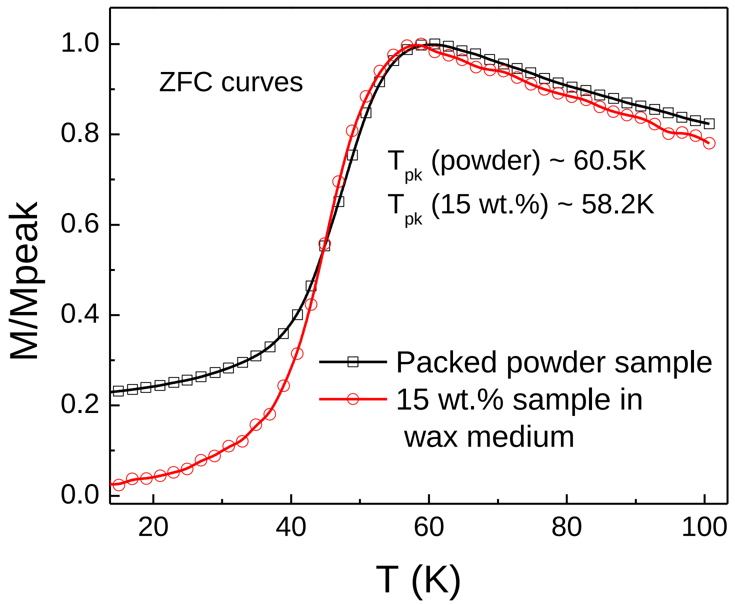

Fig. S.2 Zero-field-cooled magnetization curves for the powder sample and a dispersion of the same particles in wax, collected under a field of H_{dc} = 100 Oe and normalized to peak height.

S.2 Aging effects

A common feature of spin glasses is the aging effect of thermoremanent magnetization, i.e. the dependence of M_{TRM} on the wait time t_w at the setpoint temperature below T_{g} before switching the field off. To establish the aging behavior of the hollow nanoparticle sample, the system was rapidly cooled in a small field of 50 Oe down to 10 K. After imposing a wait time of t_w= 600s or 1800s, the field was turned off and the magnetization was recorded as a function of time.

Figure S.3(a) shows the decay of the remanent magnetization M_{TRM} normalized by the initial value M_0=M_{TRM} (t = 0). It can be seen that there is a pronounced dependence of the magnetization on tw, with a slower decrease in the sample aged for 1800s. The slow relaxation of

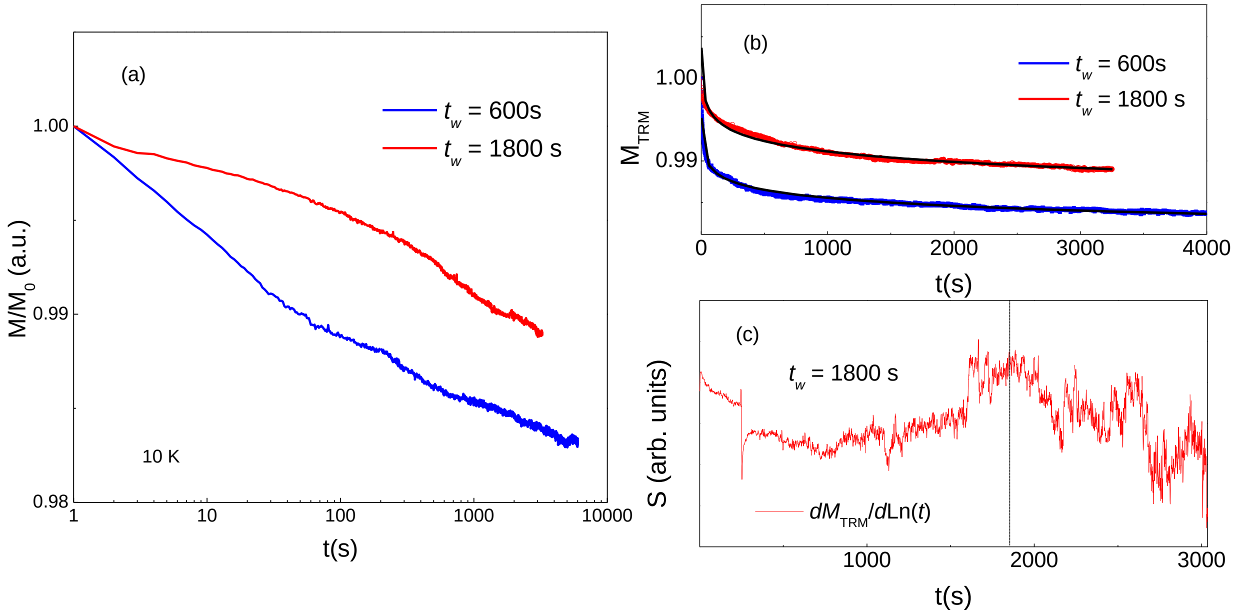

Fig. S.3 (a) Decay of normalized remanent magnetization at 10 K after a field cooling procedure and wait time t_w. (b) Best fit of M_{TRM} to Eqn. 2 (solid lines). (c) Magnetic viscosity plot for the sample aged for 1800s.

the magnetization can be roughly modeled by the expected logarithmic term superimposed on a stretched exponential function [1],

$$M_{TRM} = SH\ln(t) + M_1 + M_2\exp\left[-(t/\tau)^{1-n}\right] \quad (2)$$

with stretching exponent n ~ 0.53 consistent with spin glass behavior. From Fig. S.3(c) an inflection point at $t \sim t_w$ is also visible in the magnetic viscosity $S = dM_{TRM}/d\ln t$, a characteristic feature of spin glass aging.

[1] P. Nordblad, P. Svedlindh, L. Lundgren, and L. Sandlund, *Phys. Rev. B* **33** (1986) 645.
